# Cardiometabolic Markers Associated With Altered Fetal Growth in Mediterranean Cohort

**DOI:** 10.1111/mcn.70086

**Published:** 2025-08-06

**Authors:** Ehsan Motevalizadeh, Andrés Díaz‐López, Cristina Jardí, Cristina Rey‐Reñones, Francisco Martín‐Luján, Victoria Arija

**Affiliations:** ^1^ Research Group in Nutrition and Mental Health (NUTRISAM) Universitat Rovira i Virgili Reus Spain; ^2^ Institut d'Investigació Sanitària Pere Virgili (IISPV) Tarragona Spain; ^3^ Collaborative Group on Lifestyles, Nutrition, and Tobacco (CENIT), Tarragona‐Reus Research Support Unit Jordi Gol Primary Care Research Institute Reus Spain; ^4^ Institut d'Investigació en Atenció Primària IDIAP Jordi Gol Institut Català de la Salut (ICS) Barcelona Spain

**Keywords:** blood pressure, cardiometabolic health, fetal growth, maternal lipids, neonatal anthropometry, pregnancy

## Abstract

Cardiometabolic disturbances in pregnancy appear to be associated with inappropriate fetal growth, but evidence from uncomplicated pregnancies is still scarce and, due to varied findings, inconclusive. Moreover, most studies focus on specific markers, often measured at a single gestational time‐point. We aimed to assess the associations between maternal cardiometabolic markers, measured in early and late pregnancy, and neonatal size in a Mediterranean cohort of healthy women. Longitudinally, we analyzed 264 mother‐neonate pairs. Maternal metabolic markers (glucose, insulin resistance, triglycerides, total cholesterol, HDL‐c, LDL‐c, and blood pressure (BP)) were assessed in the first (T1) and third (T3) trimesters. Birthweight (g) and head circumference (HC, cm) were assessed in the newborns. Small (SGA, < 10th percentile) and large (LGA, > 90th percentile) for‐gestational‐age were the primary outcomes. Multivariable‐adjusted linear and logistic regressions were performed. Overall, based on weight and HC at birth, there were 10.5% and 6.4% SGA infants, while 8.1% and 16.7% were LGA, respectively. After adjustments for confounders, maternal T1 triglycerides were positively associated with birthweight (β:74.81 g per 1‐SD increment, *p* = 0.006), and higher T1 LDL‐c levels increased the risk of LGA newborns (OR:1.64 g per 1‐SD increment, *p* = 0.046). T3 diastolic‐BP was inversely associated with birthweight (β:‐86.19 g per 1‐SD increment; *p* = 0.010) and HC (β:‐0.30 g per 1‐SD increment; *p* = 0.008). High diastolic‐BP (≥ 75th percentile, 77 mmHg) was also linked to a higher risk of SGA newborns for both weight (OR:3.54, *p* = 0.022) and HC (OR:2.56 g per 1‐SD increment, *p* = 0.025). In conclusions, elevated maternal lipids in early pregnancy and diastolic BP in late pregnancy adversely impact offspring birth size, highlighting the importance of incorporating metabolic monitoring into routine prenatal care.

## Summary

- In metabolically healthy pregnant women, lipid levels and diastolic blood pressure (BP) are closely related to offspring birth size.
- Elevated maternal triglycerides and LDL‐c in early pregnancy are associated with larger birthweight and an increased risk of large‐for‐gestational‐age newborns, whereas higher diastolic‐BP during pregnancy is linked to the risk of small‐for‐gestational‐age, both in terms of birthweight and head circumference.
- Findings support that early monitoring of these cardiometabolic markers during pregnancy could improve prenatal care and help prevent adverse birth outcomes.

## 1 Introduction

Pregnancy induces profound physiological, endocrine, and metabolic adaptations to support fetal development, including dynamic shifts in glucose and lipid metabolism essential for organogenesis (Parrettini et al. 2020). However, common metabolic disturbances—such as hyperglycemia, dyslipidemia, and hypertension—can compromise intrauterine development and affect fetal growth (Catalano 2010; Mulder et al. 2024). In this context, abnormal birthweight is widely recognized as a marker of impaired fetal growth and a predictor of adverse health outcomes later in life (Belbasis et al. 2016). Being born small‐for‐gestational‐age (SGA, birthweight < 10th percentile) is associated with an increased risk of neonatal death and early neurodevelopmental impairments (Kim et al. 2024), while both SGA and large‐for‐gestational‐age (LGA, birthweight > 90th percentile) neonates—referring to those born at or beyond 33 weeks of gestation, that is, late preterm and term births—are linked to long‐term metabolic conditions such as obesity, insulin resistance (IR), type 2 diabetes, and cardiovascular disease (Belbasis et al. 2016; Johnsson et al. 2015; Risnes et al. 2011). Additionally, LGA neonates are more susceptible to delivery complications, including brachial plexus injury, shoulder dystocia, and cerebral hemorrhage (Khambalia et al. 2017). Thus, understanding the impact of unfavorable maternal metabolic profiles on inappropriate fetal growth is critical for developing early preventive strategies.

Accumulating evidence shows that elevated glucose levels at any gestational period, regardless of fasting or postprandial state, consistently are associated with higher birthweight and increased risk for LGA/macrosomia—even in women without gestational diabetes (GD) (Geurtsen et al. 2019; Guo et al. 2021; Voldner et al. 2010; Yang et al. 2023; Zhao et al. 2023; Zou et al. 2022). Nevertheless, evidence regarding maternal IR, particularly among metabolically healthy women, remains limited and inconsistent (Akinola et al. 2024; Bomba‐Opon et al. 2009; Tanaka et al. 2018; Voldner et al. 2010; Yamashita et al. 2014). Additionally, they often focus on birthweight (Bomba‐Opon et al. 2009; Tanaka et al. 2018; Yamashita et al. 2014) rather than extreme birthweight outcomes (i.e., clinically relevant outcomes like LGA and SGA). Worth noting is that altered IR in early pregnancy—a critical period for fetal development—can disrupt fetal programming and increase the offspring's risk of future metabolic disorders (Hernandez et al. 2020). Therefore, it should not be ignored.

Similarly, while a large body of literature in women from diverse racial/ethnic and cultural backgrounds has reported that elevated serum triglyceride and total cholesterol (TC) levels at any gestational time point are associated with higher birthweight and risk of LGA newborns (Adank et al. 2020; Boghossian et al. 2017; Emet et al. 2013; Gootjes et al. 2022; Jin et al. 2016; Liang et al. 2018; Okala et al. 2020; Omaña‐Guzmán et al. 2024; S. M. Zhu et al. 2022), the evidence for other lipid parameters, such as low‐density lipoprotein cholesterol (LDL‐c) and high‐density lipoprotein cholesterol (HDL‐c) levels, remains inconsistent. Only a few studies have observed associations between LDL‐c levels measured at one or two time points during gestation and neonatal size (Boghossian et al. 2017; Okala et al. 2020; S. M. Zhu et al. 2022). Findings on HDL‐c are conflicting: while some studies paradoxically link higher levels throughout pregnancy to smaller birthweight (Boghossian et al. 2017; Misra et al. 2011; H. Wang et al. 2020) and increased risk of SGA (H. Wang et al. 2020), but also to reduced risk of macrosomia in mid‐pregnancy (Clausen et al. 2005; Jin et al. 2016), others associate elevated HDL‐c in early to mid‐pregnancy with lower risk of low birthweight (LBW, < 2500 g) (Okala et al. 2020) and SGA among overweight/obese women (Bever et al. 2020). Several studies, however, report no significant associations (Adank et al. 2020; Emet et al. 2013; Gootjes et al. 2022; Jin et al. 2016; Omaña‐Guzmán et al. 2024; S. M. Zhu et al. 2022).

Several studies in Asian, European (Dutch, Swedish, and English), and North American pregnant populations have suggested that elevated maternal blood pressure (BP) during pregnancy—even below the threshold for hypertension—may negatively affect fetal growth and increase the risk of SGA (Bakker et al. 2011; Macdonald‐Wallis et al. 2014; Omaña‐Guzmán et al. 2024; Y. Zhu et al. 2019). Notably, some large population‐based cohort studies in China, focusing on preconception hypertension (*n* = 43,718) (Li et al. 2016) or gestational hypertension after 20 weeks (*n* = 16,936) (Xiong and Fraser 2004), have not supported this association.

Despite promising findings, most previous studies have assessed cardiometabolic markers at a single time point during pregnancy, neglecting the impact of longitudinal variations. Moreover, to our knowledge, no study has examined the relationship between maternal metabolic profiles and birthweight outcomes in women with uncomplicated pregnancies from the Mediterranean region, where sociodemographic and lifestyle factors may offer protection against abnormal fetal growth.

Accordingly, this study explores the associations between maternal cardiometabolic markers, assessed at two critical stages during pregnancy (i.e., early and late), and neonatal anthropometric measures and the risk of adverse birth outcomes, including SGA and LGA, while adjusting for potential maternal confounders, in healthy Mediterranean women.

## 2 Materials and Methods

### 2.1 Study Design and Participants

We conducted a prospective cohort study using data from pregnant women and their children at delivery, as part of the ECLIPSES Study (Arija et al. 2014). A total of 791 women were recruited at their first prenatal visit (≤ 12 weeks of gestation) between 2013 and 2017 from 12 sexual and reproductive health care centers (ASSIR) of the Catalan Institute of Health (ICS) in the province of Tarragona, Catalonia (Spain).

Eligible participants were healthy adult women over 18 years with ≤ 12 weeks of gestation. Major exclusion criteria included multiple pregnancies, anemia, use of iron supplements (> 10 mg/day) before 12 weeks of gestation, and any serious or chronic condition that could interfere with nutritional status or fetal development (e.g., malabsorption syndrome, diabetes, cancer, liver disease, or immunosuppressive disorders). Further details on inclusion/exclusion criteria are available elsewhere (Arija et al. 2014).

**Figure 1:**
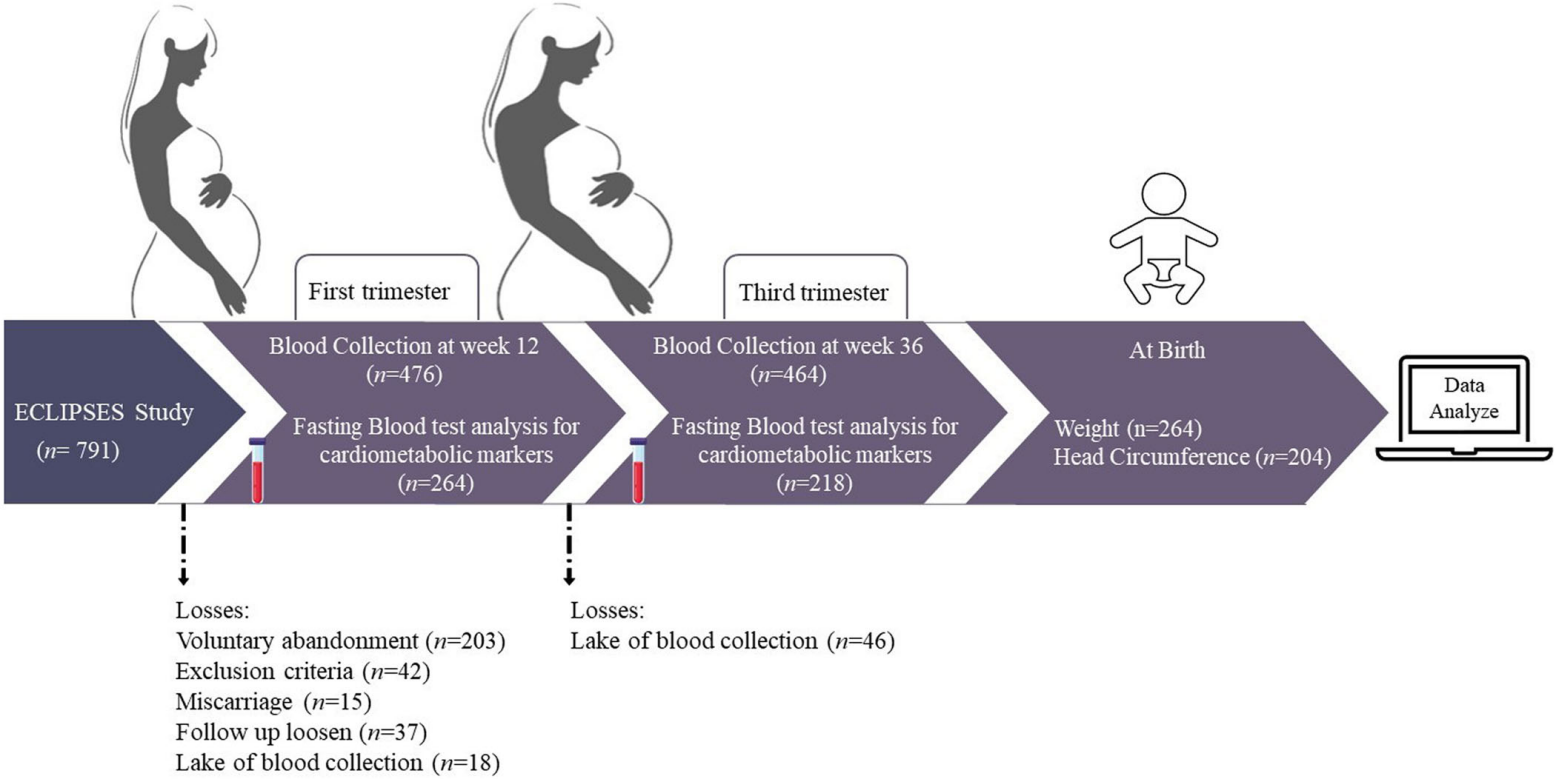
Flow chart of the study population.

From the initial 791 participants, the present analysis included 264 mother‐child pairs with available data on maternal cardiometabolic biomarkers at first (around 12 weeks of gestation) and/or third (around 36 weeks of gestation) trimester of pregnancy, as well as neonatal anthropometric measurements at birth (see Figure 1). All participants provided written informed consent. The study was conducted in accordance with the Declaration of Helsinki and was approved by the Ethics Committees of the Institut d'Investigació en Atenció Primària de Salut (IDIAP) and the Institut d'Investigació Sanitària Pere Virgili (approval ID: 118/2017, dated September 28, 2017). The ECLIPSES study is registered at www.clinicaltrialsregister.eu (ID: EUCTR‐2012‐005480‐28) and www.clinicaltrials.gov (ID: NCT03196882.

### 2.2 Newborn Anthropometric Measurements

The infant's sex and neonatal anthropometric measurements, including birthweight (grams) and head circumference (HC, cm), were obtained from the hospital delivery records and were measured immediately after birth by obstetrician or midwife following standardized procedures in accordance with the official Catalan health protocol in place at the time of data collection (Direcció General de Salut Pública de Catalunya 2008). Gestational age at birth was initially estimated by using the date of delivery minus the recalled first day of the last menstrual period (LMP) as reported by the mother at recruitment. This estimate was subsequently adjusted using crown‐rump length (CRL) measurements obtained during a standardized first‐trimester ultrasound (~ 12 week of gestation). In cases of discrepancy ≥ 5 days between the LMP‐ and ultrasound‐based dating, the ultrasound estimate was used.

The outcome variables were birthweight and HC, along with SGA and LGA at birth. Newborns below the 10th percentile for both birth anthropometric measurements assessed separately were classified as SGA, and those above the 90th percentile were classified as LGA, based on the gestational age‐ and sex‐specific growth curves from the international INTERGROWTH‐21st standards (Villar et al. 2014). It is opportune to mention that all newborns included in the present study were born at or beyond 35 weeks of gestation—3% (*n* = 8) were late preterm (≥ 35 to < 37 weeks), and 97% (*n* = 256) were term births (≥ 37–42 weeks).

### 2.3 Measurement of Maternal Cardiometabolic Markers

Maternal blood samples were collected at 12 and 36 weeks of pregnancy after an overnight fast of 8–12 h—though not all women were fasting at the time—by study nurses between 8 and 9 a.m. at the PCCs participating in the Catalunya ASSIR. After collection, the serum was separated by centrifugation and stored in 500 µL aliquots at −80°C in the Biobank until analysis. For the current study, we included only a subsample of woman who underwent fasting blood tests and had available data on the assessed cardiometabolic biomarkers, including serum glucose, insulin, and lipids (triglycerides, TC, LDL‐c, and HDL‐c). Serum glucose, TC, HDL‐c, and triglycerides concentrations were measured using standard enzymatic automated methods, with intra‐ and interassay coefficients of variation (CVs) below 2.2% for all. LDL‐c was calculated using the Friedewald formula: LDL‐c= TC ‐ HDL‐c ‐ (triglycerides/5). Serum insulin levels were measured by a chemiluminescent immunoassay method on an ADVIA Centaur analyzer using a commercial kit (ADVIA Centaur IRI, Siemens Healthcare Diagnostics Inc., Tarrytown, NY, USA). The lower and upper detection limits were 0.5 and 300 mUI/L, respectively, with intra‐ and interassay CVs ranging from 3.3% to 4.6% and 2.6%–5.9%, respectively. All measurements were conducted at the ICS Camp de Tarragona‐Terres de l'Ebre accredited laboratory, Joan XXIII University Hospital in Tarragona. To minimize inter‐batch variation, the samples were thawed and assayed simultaneously at the end of the study.

IR was estimated using the HOMA‐IR index, calculated as HOMA‐IR = [fasting glucose (mmol/L) × fasting insulin (μIU/mL)]./22.5. BP was measured in both trimesters by trained personnel at the PCCs using an automated digital monitor (Omron HEM‐705CP), following standardized procedures recommended for clinical practice (seated position, after 5 min of rest, with the appropriate cuff size).

### 2.4 Covariates

Midwives and nutritionists collected data in the first trimester (week 12) through personal interviews and specific questionnaires, covering demographics (age, socioeconomic status (SES) and education), health behaviors (physical activity (PA), smoking, and diet), and obstetric history (parity (nulliparous vs. multiparous). SES was calculated by combining information on occupational status, classified in accordance with the Catalan classification of occupations (CCO‐2011), and educational level (Arija et al. 2014). It was then classified as low, middle, or high. The educational level was classified as low (primary school or less), medium (secondary studies), and high (university studies or above). Pregnancy planning was assessed with the question “Was this pregnancy planned? (yes/no)”: a “yes” response defined it as planned, meaning intentionally desired at conception, while a “no” response defined it as unplanned, including mistimed and/or unwanted pregnancies.

Overall diet quality was assessed using the relative Mediterranean Diet (rMedDiet) score, based on the intake of nine food groups from a 45‐item self‐administered food frequency questionnaire previously validated in this population (Jardí et al. 2019). The rMedDiet score (ranging from 0 to 18 points) was categorized into tertiles (T1: < 9, T2: 9–12, T3: ≥ 12). PA was assessed using a shortened version of the International PA Questionnaire (IPAQ‐S) (Craig et al. 2003), and categorized into tertiles based on weekly metabolic equivalents (METs‐min/week) (T1: < 1070, T2: 1070‐3336, T3: ≥ 3336). The Fagerström questionnaire (Heatherton et al. 1991) was used to assess smoking, and women were classified as current smokers or non‐smokers (former and never smokers). Alcohol consumption was assessed as “yes” or “no.”

Body mass index (BMI) was calculated from weight and measurements taken at enrollment and during each trimester, and women were classified into three groups: normal weight (BMI < 24.9 kg/m^2^), overweight (BMI 25.0–29.9 kg/m^2^), and obesity (BMI ≥ 30.0 kg/m^2^) (WHO 2000). Total gestational weight gain (GWG) was calculated based on BMI and classified as insufficient, adequate, and excessive according to the 2009 Institute of Medicine standards.

### 2.5 Statistical Analysis

Data were analyzed using software SPSS (version 29.0). Descriptive statistics were presented as mean ± SD for continuous variables or number (%) for categorical variables. Maternal cardiometabolic markers (triglycerides, TC, LDL‐c, HDL‐c, glucose, HOMA‐IR, systolic BP, and diastolic BP) were divided into quartiles, with the lowest quartile as the reference.

Multivariable linear regression analyses were performed to explore the association between each maternal cardiometabolic marker separately as continuous (per 1‐SD increase) exposure variables in both the first and third trimesters and each newborn anthropometric measurement as a continuous outcome (birthweight and HC). Predefined confounders based on prior literature included maternal age, BMI, GWG, education, social class, smoking status, PA, rMedDiet score, planned pregnancy, parity, and infant sex. Estimates were reported as β coefficients with 95% confidence intervals (CIs). A test for linear trend was calculated by treating ordinal categorical exposure variable as a continuous variable.

Additionally, adjusted logistic regression models estimated odds ratios (ORs) and 95% CIs for the risk of SGA and LGA at birth linked to each maternal cardiometabolic marker (in separate models), assessed as both continuous (per 1‐SD increase) and categorical exposure variables (normal‐low < 75th (reference) vs. high ≥ 75th percentile). These analyses included the same covariates as the linear models. Statistical significance was set at *p* < 0.05.

### 2.6 Ethics Statement

The ECLIPSES study was registered both in ClinicalTrials.gov (identification number NCT03196882) and the EU Clinical Trials Register (EUCTR‐2012‐005480‐28). The study was approved by the Ethical Committee of the Jordi Gol Institute for Primary Care Research and the Pere Virgili Institute for Health Research Research (approval ID: 118/2017. Date: September 28, 2017) and complied with the tenets of the Helsinki declaration.

## 3 Results

The study sample consisted of 264 mothers and their babies (51.1% boys). Table 1 presents the general characteristics of the mothers and their newborns. Overall, the average maternal age was 29.6 years, with a mean BMI of 24.1 kg/m². Among the women, 32% held a university degree, 19% were classified as high SES, and 14% reported smoking during pregnancy. The anthropometric measurements of the newborns at birth were within normal ranges, with a mean weight of 3316.9 ± 426.8 g and a mean HC of 34.5 ± 1.3 cm. The average gestational age was 39.6 ± 2.2 weeks.

**Table 1:** General maternal and child characteristics: sociodemographic, lifestyle and anthropometric data (n = 264).

Maternal characteristics	Summary statistics
Age (years)	29.64 ± 4.71
Age categories (years)	
< 25	40 (15.2)
25–29	73 (27.7)
≥ 30	151 (57.2)
Weight (kg)	63.26 ± 9.65
BMI (kg/m^2^)	24.12 ± 3.53
BMI categories	
18.5–24.9 (normal weight)	169 (64.0)
25.0–29.9 (overweight)	81 (30.7)
≥ 30 (obesity)	14 (5.3)
GWG (kg)	10.56 ± 3.69
IOM GWG recommendations^[Link]^, ^a^	
Insufficient	114 (43.2)
Adequate	103 (39.0)
Excessive	47 (17.8)
Educational level	
Low (primary or below)	83 (31.4)
Medium (secondary)	97 (36.7)
High (university or above)	84 (31.8)
Social class	
Low	35 (13.3)
Medium	180 (68.2)
High	49 (18.6)
Smoking status	
Never/Former smoker	227 (86.0)
Current smoker	37 (14.0)
Alcohol consumption	
No	222 (87.1)
Yes	33 (12.9)
Physical Activity (METs‐min/week)	
T1 (< 1070)	90 (34.1)
T2 (1070‐3336)	114 (43.2)
T3 (≥ 3336)	60 (22.7)
rMedDiet score (point)	
T1 (< 9)	92 (36.1)
T2 (9–12)	107 (42.0)
T3 (≥ 12)	56 (22.0)
Planned pregnancy	
No	62 (23.5)
Yes	202 (76.5)
Parity	
No	112 (42.4)
Yes	152 (57.6)
Newborn characteristics	
Infant's sex	
Female	129 (48.9)
Male	135 (51.1)
Birth weight (g)	3316.95 ± 426.83
Birth HC (cm)^b^	34.54 ± 1.30
GA at delivery (weeks)	39.76 ± 1.28

*Note:* Values are expressed as means ± standard deviation or number (%, percentage).

Abbreviations: BMI, body mass index; GA, gestational age; GWG, gestational weight gain; HC, head circumference; IOM, Institute of Medicine; METs, metabolic equivalents; rMedDiet, Mediterranean diet; T, tertile.

^a^ Recommendations for GWG according to IOM guidelines are: initial BMI < 18.5 kg/m^2^, total weight gain 12.5–18 kg; BMI 18.5–24.9 kg/m^2^, total weight gain 11.5–16 kg; BMI 25.0–29.9 kg/m2, total weight gain 7–11.5 kg; and BMI ≥ 30 kg/m^2^ total weight gain 5–9 kg.

^b^ *n* = 204.

Table 2 displays the mean values of lipids, glucose, HOMA‐IR, and blood pressure measured during early and late pregnancy. Notably, all indicators except glucose showed an increasing trend in the third trimester, while remaining within clinically acceptable ranges.

**Table 2:** Maternal cardiometabolic markers levels during pregnancy according to the different quartile.

		Quartiles			
Cardiometabolic markers	All	Q1	Q2	Q3	Q4
First trimester (*n* = 264)	mean ± SD	mean ± SD	mean ± SD	mean ± SD	mean ± SD
Triglycerides (mg/dL)	89.23 ± 37.16	54.30 ± 7.50	71.71 ± 4.74	91.09 ± 6.59	140.61 ± 34.46
Total cholesterol (mg/dL)	167.28 ± 34.01	132.97 ± 8.84	154.14 ± 5.07	171.94 ± 6.46	211.26 ± 34.73
LDL‐c (mg/dL)	88.18 ± 25.49	63.56 ± 6.60	78.13 ± 3.76	90.78 ± 3.80	120.23 ± 28.09
HDL‐c (mg/dL)	61.28 ± 13.04	46.95 ± 4.05	56.20 ± 1.97	64.12 ± 2.96	79.17 ± 8.74
Glucose (mg/dL)	70.23 ± 10.73	57.87 ± 8.99	67.95 ± 1.45	72.95 ± 1.45	82.68 ± 6.71
HOMA‐IR	1.63 ± 1.20	0.65 ± 0.17	1.10 ± 0.12	1.52 ± 0.15	3.26 ± 1.35
SBP (mm Hg)	112.34 ± 11.85	98.99 ± 5.72	109.17 ± 1.90	116.88 ± 2.46	129.04 ± 5.81
DBP (mm Hg)	66.44 ± 7.75	57.49 ± 3.25	64.72 ± 1.69	69.66 ± 1.01	77.09 ± 4.13
Third trimester (*n* = 218)					
Triglycerides (mg/dL)	**188.86** ± 76.93*	90.4 ± 26.32	166.82 ± 17.23	214.77 ± 15.26	286.93 ± 43.06
Total cholesterol (mg/dL)	**237.81** ± 45.24**	185.70 ± 18.73	219.28 ± 7.85	248.53 ± 9.04	299.88 ± 27.51
LDL‐c (mg/dL)	**131.19** ± 35.85**	91.96 ± 14.78	115.69 ± 5.54	137.47 ± 7.44	180.40 ± 24.82
HDL‐c (mg/dL)	**66.33** ± 14.13**	50.48 ± 5.81	62.00 ± 2.17	70.29 ± 3.13	86.91 ± 10.51
Glucose (mg/dL)	**67.40** ± 10.07*	55.23 ± 7.56	65.19 ± 1.46	70.33 ± 1.80	80.02 ± 5.61
HOMA‐IR	1.84 ± 1.63	0.67 ± 0.21	1.23 ± 0.15	1.69 ± 0.15	3.78 ± 2.27
SBP (mm Hg)	**115.82** ± 11.15**	101.52 ± 4.39	112.56 ± 2.02	120.66 ± 2.69	130.59 ± 4.05
DBP (mm Hg)	**70.66** ± 7.94**	60.82 ± 3.42	68.38 ± 1.52	73.73 ± 1.80	81.39 ± 3.57

*Note:* Values are expressed as means ± standard deviation. Reference values in the first/third trimester: triglycerides 40–159/131–453 mg/dL; total cholesterol 141–210/219–349 mg/dL; LDL‐c 60–153/101–224 mg/dL, HDL‐c 40–78/48–87 mg/dL (Abbassi‐Ghanavati et al. 2009); glucose 81.59/79.52 mg/dL; HOMA‐IR 1.25/1.81 (Sonagra et al. 2014); SBP ‐ DBP 112.1– 65.4/116.0 – 70.0 (Macdonald‐Wallis et al. 2015). Values in bold indicate statistically significant estimates; * denotes *p* < 0.05 and ** denotes *p* < 0.001. These values were obtained by comparison with the first trimester using the paired Student's *t*‐test.

Abbreviations: DBP, diastolic blood pressure; HDL‐c, high density lipoprotein cholesterol; HOMA‐IR, homeostatic model assessment for insulin resistance; LDL‐c, low density lipoprotein cholesterol; SBP, systolic blood pressure; SD, standard deviation.

Tables 3 and 4 present multivariable‐adjusted linear regression results examining associations between each cardiometabolic marker in the first and third trimesters and newborn anthropometric outcomes. After adjusting for covariates, triglyceride levels in the first trimester were positively associated with birthweight, both continuously (β: 74.81 g per 1‐SD increase; 95% CI: 21.81, 127.82, *p* = 0.006) and categorically (β_Q4 (≥ 105 mg/dL) vs. Q1 (≤ 64 mg/dL, reference)_): 175.98 g; 95% CI: 24.58, 327.38, p‐trend = 0.029). No significant associations were found between other cardiometabolic parameters and newborn anthropometry measures in the first trimester (Table 3).

**Table 3:** Multivariable‐adjusted linear regression models for the associations of maternal cardiometabolic markers in the first trimester and birth outcomes.

	Birth weight (g)		Birth HC (cm)	
Cardiometabolic markers	β (95% CI)	*p*	β (95% CI)	*p*
Triglycerides (mg/dL)				
Continuous (per 1‐SD increase)	**74.81 (21.81, 127.82)**	0.006*	0.10 (−0.10, 0.29)	0.343
Q1	0.0 (ref.)		0.0 (ref.)	
Q2	20.94 (−127.27, 169.15)	0.781	−0.19 (−0.71, 0.32)	0.455
Q3	39.66 (−106.02, 185.33)	0.592	−0.19 (−0.71, 0.31)	0.445
Q4	**175.98 (24.58, 327.38)**	0.023*	0.16 (−0.39, 0.74)	0.539
* p*‐trend	0.029*		0.675	
Total cholesterol (mg/dL)				
Continuous (per 1‐SD increase)	51.17 (−1.55, 103.89)	0.057	0.05 (−0.14, 0.24)	0.616
Q1	0.0 (ref.)		0.0 (ref.)	
Q2	51.74 (−97.52, 201.01)	0.495	0.22 (−0.29, 0.73)	0.400
Q3	−14.42 (−167.82, 138.98)	0.853	0.09 (−0.46, 0.64)	0.755
Q4	75.18 (−76.41, 226.79)	0.330	0.06 (−0.48, 0.59)	0.841
* p*‐trend	0.655		0.993	
LDL‐c (mg/dL)				
Continuous (per 1‐SD increase)	35.58 (−17.87, 89.04)	0.191	0.04 (−0.16, 0.23)	0.719
Q1	0.0 (ref.)		0.0 (ref.)	
Q2	31.67 (−117.43, 180.77)	0.676	0.22 (−0.29, 0.74)	0.392
Q3	46.25 (−106.53, 199.04)	0.551	0.12 (−0.41, 0.65)	0.661
Q4	29.73 (−124.72, 184.18)	0.705	0.03 (−0.49, 0.56)	0.902
* p*‐trend	0.666		0.956	
HDL‐c (mg/dL)				
Continuous (per 1‐SD increase)	22.90 (−30.47, 76.27)	0.399	0.00 (−0.18, 0.19)	0.977
Q1	0.0 (ref.)		0.0 (ref.)	
Q2	105.09 (−46.42, 256.58)	0.173	0.54 (−0.01, 1.09)	0.056
Q3	104.50 (−43.37, 252.37)	0.165	0.29 (−0.24, 0.82)	0.284
Q4	114.68 (−36.11, 265.48)	0.135	0.19 (−0.35, 0.74)	0.478
*p*‐trend	0.147		0.701	
Glucose (mg/dL)				
Continuous (per 1‐SD increase)	14.81 (−37.09, 66.72)	0.574	−0.10 (−0.29, 0.09)	0.288
Q1	0.0 (ref.)		0.0 (ref.)	
Q2	21.85 (−125.97, 171.65)	0.774	−0.28 (−0.81, 0.25)	0.298
Q3	97.69 (−52.56, 247.93)	0.201	0.16 (−0.37, 0.69)	0.551
Q4	27.47 (−122.23, 177.17)	0.718	−0.32 (0.83, 0.20)	0.230
* p*‐trend	0.515		0.528	
HOMA‐IR				
Continuous (per 1‐SD increase)	27.70 (−26.01, 81.42)	0.311	0.07 (−0.14, 0.29)	0.507
Q1	0.0 (ref.)		0.0 (ref.)	
Q2	55.07 (−95.94, 206.08)	0.473	−0.16 (−0.68, 0.37)	0.550
Q3	129.13 (−24.81, 283.07)	0.100	−0.21 (−0.75, 0.34)	0.451
Q4	67.83 (−85.12, 220.78)	0.383	−0.21 (−0.76, 0.34)	0.457
* p*‐trend	0.285		0.449	
SBP (mm Hg)				
Continuous (per 1‐SD increase)	6.22 (−50.21, 62.66)	0.828	−0.07 (−0.26, 0.13)	0.481
Q1	0.0 (ref.)		0.0 (ref.)	
Q2	33.78 (−116.72, 184.28)	0.659	−0.06 (−0.58, 0.45)	0.808
Q3	64.63 (−76.04, 205.30)	0.366	0.09 (−0.40, 0.58)	0.716
Q4	−20.52 (−175.19, 134.17)	0794	−0.33 (−0.88, 0.22)	0.243
* p*‐trend	0.955		0.441	
DBP (mm Hg)				
Continuous (per 1‐SD increase)	6.95 (−47.69, 61.59)	0.802	−0.02 (−0.20, 0.16)	0.816
Q1	0.0 (ref.)		0.0 (ref.)	
Q2	120.07 (−21.98, 262.12)	0.097	0.370 (−0.12, 0.86)	0.136
Q3	42.57 (−103.65, 188.79)	0.567	0.03 (−0.49, 0.54)	0.920
Q4	−6.61 (−158.25, 145.03)	0.932	−0.04 (−0.56, 0.49)	0.892
* p*‐trend	0.812		0.730	

*Note:* Linear regression models were used to calculate the β coefficient (β) and 95% confidence interval (95% CI). For continuous exposure variables, β denotes the change in birthweight (g) or HC (cm) associated with a 1‐SD increase in the exposure. Adjusted for age categories (< 25 (ref.), 25–29, ≥ 30 years), physical activity tertile (T1: ≤ 1070 (ref.), T2:1071‐3335, T3: ≥ 3336 METs‐min/week), Mediterranean diet score tertile (T1: ≤ 8 (ref.), T2: 9‐11, T3: ≥ 12 points), GWG (insufficient (ref), adequate, excessive), BMI categories (normal weight (ref.), overweight/obesity), parity (nulliparous (ref.), multiparous), educational level (low/medium (ref.), high), smoking status (never/former smoker (ref.), current smoker), planned pregnancy (no (ref.), yes), social class (low/medium (ref.), high), sex infant. Values in bold indicate statistically significant estimates; * denotes *p* < 0.05. The *p*‐value for the trend was calculated by treating ordinal categorical exposure variable as a continuous variable.

Abbreviations: DBP, diastolic blood pressure; HC, head circumference; HDL‐c, high density lipoprotein cholesterol; HOMA‐IR, homeostatic model assessment for insulin resistance; LDL‐c, low density lipoprotein cholesterol; SBP, systolic blood pressure; SD, standard deviation.

**Table 4:** Multivariable‐adjusted linear regression models for the associations of maternal cardiometabolic markers in the third trimester, and birth outcomes.

	Birth weight (g)		Birth HC (cm)	
Cardiometabolic markers	β (95% CI)	*p*	β (95% CI)	*p*
Triglycerides (mg/dL)				
Continuous (per 1‐SD increase)	−2.13 (−0.60, 0.56)	0.943	−0.02 (−0.23, 0.18)	0.825
Q1	0.0 (ref.)		0.0 (ref.)	
Q2	−15.25 (−175.25, 144.77)	0.851	−0.05 (−0.62, 0.52)	0.856
Q3	−83.72 (−243.37, 75.93)	0.302	−0.18 (−0.74, 0.38)	0.523
Q4	28.89 (−134.33, 192.12)	0.727	−0.06 (−0.64, 0.51)	0.833
* p*‐trend	0.983		0.702	
Total cholesterol (mg/dL)				
Continuous (per 1‐SD increase)	−36.83 (−92.84, 19.18)	0.196	−0.14 (−0.34, 0.05)	0.151
Q1	0.0 (ref.)		0.0 (ref.)	
Q2	−57.54 (−213.99, 98.92)	0.469	−0.36 (−0.92, 0.19)	0.196
Q3	−28.93 (−184.34, 126.49)	0.714	−0.24 (−0.80, 0.33)	0.407
Q4	−151.24 (−315.21, 12.73)	0.070	−0.51 (−1.09, 0.07)	0.085
* p*‐trend	0.119		0.124	
LDL‐c (mg/dL)				
Continuous (per 1‐SD increase)	−36.83 (−90.58, 16.91)	0.178	−0.11 (−0.29, 0.08)	0.249
Q1	0.0 (ref.)		0.0 (ref.)	
Q2	−26.00 (−177.55, 125.55)	0.736	−0.01 (−0.55, 0.52)	0.962
Q3	8.60 (−142.77, 159.98)	0.911	−0.02 (−0.56, 0.52)	0.945
Q4	−79.65 (−236.04, 76.73)	0.317	−0.14 (−0.69, 0.42)	0.619
* p*‐trend	0.428		0.629	
HDL‐c (mg/dL)				
Continuous (per 1‐SD increase)	−8.18 (−64.86, 48.51)	0.776	−0.03 (−0.17, 0.22)	0.786
Q1	0.0 (ref.)		0.0 (ref.)	
Q2	−34.79 (−188.74, 119.15)	0.656	−0.07 (−0.63, 0.49)	0.816
Q3	−105.136 (−263.67, 53.40)	0.192	−0.07 (−0.62, 0.49)	0.808
Q4	−18.92 (−187.25, 149.41)	0.825	0.20 (−0.39, 0.79)	0.504
* p*‐trend	0.556		0.584	
Glucose (mg/dL)				
Continuous	41.86 (−14.57, 98.29)	0.145	0.04 (−0.17, 0.25)	0.714
Q1	0.0 (ref.)		0.0 (ref.)	
Q2	−4.55 (−170.84, 161.74)	0.957	−0.39 (−0.97, 0.18)	0.176
Q3	83.02 (−74.73, 240.77)	0.301	−0.03 (−0.61, 0.56)	0.936
Q4	67.51 (−98.07, 233.08)	0.422	−0.19 (−0.78, 0.39)	0.504
* p*‐trend	0.251		0.846	
HOMA‐IR				
Continuous (per 1‐SD increase)	12.46 (−52.27, 77.20)	0.705	−0.07 (−0.33, 0.19)	0.585
Q1	0.0 (ref.)		0.0 (ref.)	
Q2	10.89 (−153.82, 175.59)	0.896	0.11 (−0.47, 0.68)	0.717
Q3	−18.23 (−181.29, 144.84)	0.826	−0.23 (−0.79, 0.33)	0.415
Q4	−39.38 (−206.07, 127.30)	0.642	−0.36 (−0.98, 0.26)	0.252
* p*‐trend	0.576		0.153	
SBP (mm Hg)				
Continuous (per 1‐SD increase)	−54.51 (−122, 13.85)	0.117	−0.16 (−0.39, 0.08)	0.184
Q1	0.0 (ref.)		0.0 (ref.)	
Q2	−55.26 (−231.09, 120.65)	0.536	−0.02 (−0.61, 0.57)	0.945
Q3	−108.09 (−294.39, 78.14)	0.253	−0.19 (−0.83, 0.45)	0.551
Q4	−133.18 (−325.65, 59.28)	0.174	−0.31 (−0.98, 0.36)	0.365
* p*‐trend	0.143		0.318	
DBP (mm Hg)				
Continuous (per 1‐SD increase)	**−86.19 (−151.62, −20.76)**	0.010*	**−0.30 (−0.52, −0.08)**	0.008*
Q1	0.0 (ref.)		0.0 (ref.)	
Q2	−91.75 (−270.44, 86.94)	0.312	−0.42 (−1.02, 0.18)	0.165
Q3	−73.67 (−253.24, 105.89)	0.419	**−0.63 (−1.26, −0.01)**	0.047*
Q4	**−245.49 (−431.54, −59.45)**	0.010*	**−0.87 (−1.49, −0.24)**	0.007*
* p*‐trend	0.018*		0.005*	

*Note:* Linear regression models were used to calculate the β coefficient (β) and 95% confidence interval (95% CI). For continuous exposure variables, β denotes the change in birthweight (g) or HC (cm) associated with a 1‐SD increase in the exposure. Adjusted for age categories (< 25 (ref.), 25‐29, ≥ 30 years), physical activity tertile (T1: ≤ 1070 (ref.), T2:1071‐3335, T3: ≥ 3336 METs‐min/week), Mediterranean diet score tertile (T1: ≤ 8 (ref.), T2: 9‐11, T3: ≥ 12 points), GWG (insufficient (ref), adequate, excessive), BMI categories (normal weight (ref.), overweight/obesity), parity (nulliparous (ref.), multiparous), educational level (low/medium (ref.), high), smoking status (never/former smoker (ref.), current smoker), planned pregnancy (no (ref.), yes), social class (low/medium (ref.), high), sex infant. Values in bold indicate statistically significant estimates; * denotes *p* < 0.05. The *p*‐value for the trend was calculated by treating ordinal categorical exposure variable as a continuous variable.

Abbreviations: DBP, diastolic blood pressure; HC, head circumference; HDL‐c, high density lipoprotein cholesterol; HOMA‐IR, homeostatic model assessment for insulin resistance; LDL‐c, low density lipoprotein cholesterol; SBP, systolic blood pressure; SD, standard deviation.

In the third trimester, diastolic‐BP was inversely associated with birthweight, both in continuous analysis (β: −86.19 g per 1‐SD increase; 95% CI: −151.62, −20.76; *p* = 0.010) and categorical analysis (β_Q4 (≥ 77 mmHg) vs. Q1 (≤ 65 mmHg, reference)_): −245.49 cm; 95% CI: −431.54, −59.45, p‐trend = 0.018). Similarly, diastolic‐BP showed a negative association with birth HC in the continuous model (β: −0.30; cm; 95% CI: −0.52, −0.08, *p* = 0.008). Compared to newborns of mothers in the lowest quartile, those of mothers in the third (β_Q3 (71–76 mmHg) vs. Q1 (≤ 65 mmHg, reference)_): −0.63 cm; 95% CI: −1.26, −0.01, *p* = 0.047) and fourth quartiles (β_Q4 (≥ 77 mmHg) vs. Q1 (≤ 65 mmHg, reference)_): −0.87 cm; 95% CI: −1.49, −0.24, *p* = 0.007) of diastolic‐BP had substantially lower HC (Table 4).

Overall, 10.5% (*n* = 27) and 6.4% (*n* = 13) of infants were classified as SGA based on birthweight and HC, whereas 8.1% (*n* = 21) and 16.7% (*n* = 34) were classified as LGA based on birthweight and HC, respectively. After adjusting for covariates, first‐trimester triglyceride levels were associated with a decreased risk of birthweight‐based SGA (OR: 0.38 per 1‐SD increase; 95% CI: 0.19, 0.79; *p* = 0.010). The OR for triglycerides in the ≥ 75th percentile (≥ 105 mg/dL) was 0.21 (95% CI: 0.04, 0.95; *p* = 0.043). At the same time, LDL‐c levels were associated with an elevated risk of LGA based on birthweight (OR: 1.64 per 1‐SD increase; 95% CI: 1.08, 2.48; *p* = 0.046), whereas diastolic‐BP levels were associated with SGA based on HC (OR: 2.53 per 1‐SD increase; 95% CI: 1.20, 5.34; *p* = 0.015) (Table 5).

**Table 5:** Multivariable‐adjusted odds ratio and 95% confidence interval, for the associations of maternal cardiometabolic markers in the first trimester, and birth outcomes.

	Birth weight				Birth HC			
	SGA		LGA		SGA		LGA	
Cardiometabolic markers	N (%)	OR (95% CI)	N (%)	OR (95% CI)	N (%)	OR (95% CI)	N (%)	OR (95% CI)
Triglycerides (mg/dL)								
Continuous (per 1‐SD increase)	27 (10.5)	0.38 (0.19, 0.79)*	21 (8.1)	1.32 (0.84, 2.07)	13 (6.4)	0.63 (0.22, 1.76)	34 (16.7)	1.02 (0.66, 1.55)
Normal (< 75th Pctl)	25 (12.9)	1 Ref.	12 (6.2)	1 Ref.	12 (7.6)	1 Ref.	25 (15.8)	1 Ref.
High (≥ 75th Pctl)	2 (3.1)	0.21 (0.04, 0.95)*	9 (14.1)	2.49 (0.85, 7.32)	1 (2.2)	0.43 (0.05, 4.05)	9 (19.6)	0.75 (0.27, 2.03)
Total cholesterol (mg/dL)								
Continuous (per 1‐SD increase)	27 (10.5)	0.67 (0.39, 1.16)	21 (8.1)	1.47 (0.96, 2.24)	13 (6.4)	0.70 (0.30, 1.65)	34 (16.7)	1.05 (0.69, 1.59)
Normal (< 75th Pctl)	20 (10.6)	1 Ref.	12 (6.4)	1 Ref.	11 (7.3)	1 Ref.	26 (17.3)	1 Ref.
High (≥ 75th Pctl)	7 (10.0)	0.85 (0.32, 2.25)	9 (12.9)	2.69 (0.94, 7.75)	2 (3.7)	0.59 (0.10, 3.46)	8 (14.8)	0.68 (0.26, 1.75)
LDL‐c (mg/dL)								
Continuous (per 1‐SD increase)	27 (10.5)	0.79 (0.47, 1.34)	21 (8.1)	1.64 (1.08, 2.48)*	13 (6.4)	0.67 (0.26, 1.77)	34 (16.7)	1.10 (0.73, 1.64)
Normal (< 75th Pctl)	20 (10.4)	1 Ref.	14 (7.3)	1 Ref.	11 (7.2)	1 Ref.	26 (17.0)	1 Ref.
High (≥ 75th Pctl)	7 (10.8)	0.82 (0.29, 2.32)	7 (10.8)	1.55 (0.52, 4.61)	2 (3.9)	1.04 (0.16, 6.66)	8 (15.7)	0.59 (0.22, 1.58)
HDL‐c (mg/dL)								
Continuous (per 1‐SD increase)	27 (10.5)	0.87 (0.55, 1.37)	21 (8.1)	0.75 (0.42, 1.31)	13 (6.4)	0.96 (0.51, 1.81)	34 (16.7)	0.93 (0.61, 1.40)
Normal (< 75th Pctl)	21 (11.1)	1 Ref.	17 (8.9)	1 Ref.	10 (6.8)	1 Ref.	26 (17.6)	1 Ref.
High (≥ 75th Pctl)	6 (8.8)	0.74 (0.26, 2.14)	4 (5.9)	0.60 (0.17, 2.13)	3 (5.4)	0.88 (0.19, 4.16)	8 (14.3)	1.12 (0.42, 2.99)
Glucose (mg/dL)								
Continuous (per 1‐SD increase)	27 (10.5)	1.03 (0.67, 1.60)	21 (8.1)	0.85 (0.54, 1.34)	13 (6.4)	1.41 (0.68, 2.95)	34 (16.7)	0.89 (0.61, 1.31)
Normal (< 75th Pctl)	18 (9.3)	1 Ref.	16 (8.3)	1 Ref.	9 (6.0)	1 Ref.	26 (17.3)	1 Ref.
High (≥ 75th Pctl)	9 (13.8)	1.76 (0.69, 4.52)	5 (7.7)	0.87 (0.27, 2.79)	4 (7.4)	1.37 (0.33, 5.71)	8 (14.8)	0.67 (0.26, 1.75)
HOMA‐IR								
Continuous (per 1‐SD increase)	27 (10.5)	0.83 (0.52, 1.34)	21 (8.1)	0.78 (0.40, 1.51)	13 (6.4)	0.86 (0.38, 1.92)	34 (16.7)	0.96 (0.59, 1.55)
Normal (< 75th Pctl)	20 (10.4)	1 Ref.	16 (8.3)	1 Ref.	10 (6.4)	1 Ref.	25 (15.9)	1 Ref.
High (≥ 75th Pctl)	7 (10.8)	1.02 (0.38, 2.76)	5 (7.7)	0.97 (0.29, 3.17)	3 (6.4)	1.52 (0.27, 5.87)	9 (19.1)	0.77 (0.28, 2.09)
SBP (mm Hg)								
Continuous (per 1‐SD increase)	27 (10.5)	0.78 (0.48, 1.28)	21 (8.1)	1.07 (0.62, 1.84)	13 (6.4)	1.26 (0.66, 2.40)	34 (16.7)	0.75 (0.47, 1.18)
Normal (< 75th Pctl)	22 (11.8)	1 Ref.	14 (7.5)	1 Ref.	9 (5.9)	1 Ref.	25 (16.3)	1 Ref.
High (≥ 75th Pctl)	5 (7.0)	0.56 (0.19, 1.67)	7 (9.9)	1.04 (0.35, 3.14)	4 (7.8)	1.74 (0.42, 7.12)	9 (17.6)	0.87 (0.32, 2.36)
DBP (mm Hg)								
Continuous (per 1‐SD increase)	27 (10.5)	1.08 (0.69, 1.69)	21 (8.1)	1.08 (0.64, 1.81)	13 (6.4)	2.53 (1.20, 5.34)*	34 (16.7)	1.08 (0.71, 1.64)
Normal (< 75th Pctl)	18 (9.7)	1 Ref.	15 (8.1)	1 Ref.	7 (4.6)	1 Ref.	27 (17.9)	1 Ref.
High (≥ 75th Pctl)	9 (12.5)	1.08 (0.43, 2.73)	6 (8.3)	0.87 (0.29, 2.63)	6 (11.3)	3.28 (0.84, 12.76)	7 (13.2)	0.62 (0.23, 1.68)

*Note:* Logistic regression models were used to calculate the Odds Ratio (OR) and 95% confidence interval (95% CI). Adjusted for age categories (< 25 (ref.), 25‐29, ≥ 30 years), physical activity tertile (T1: ≤ 1070 (ref.), T2:1071‐3335, T3: ≥ 3336 METs‐min/week), Mediterranean diet score tertile (T1: ≤ 8 (ref.), T2: 9‐11, T3: ≥ 12 points), GWG (insufficient (ref), adequate, excessive), BMI categories (normal weight (ref.), overweight/obesity), parity (nulliparous (ref.), multiparous), educational level (low/medium (ref.), high), smoking status (never/former smoker (ref.), current smoker), planned pregnancy (no (ref.), yes), social class (low/medium (ref.), high), sex infant. Values in bold indicate statistically significant estimates; * denotes *p* < 0.05.

Abbreviations: DBP, diastolic blood pressure; HC, head circumference; HDL‐c, high density lipoprotein cholesterol; HOMA‐IR, homeostatic model assessment for insulin resistance; LDL‐c, low density lipoprotein cholesterol; SBP, systolic blood pressure; SD, standard deviation.

In the third trimester, diastolic‐BP levels remained significantly associated with an increased likelihood of HC‐based SGA newborns (OR: 2.56 per 1‐SD increase; 95% CI: 1.13, 5.81; *p* = 0.025) and birthweight‐based SGA (OR: 2.09 per 1‐SD increase; 95% CI: 1.22, 3.58; *p* = 0.007). Moreover, infants born to mothers with diastolic‐BP at or above the 75th percentile (≥ 77 mmHg) had a higher risk of birthweight‐SGA (OR: 3.54; 95% CI: 1.20, 10.42; *p* = 0.022). There were no relationships between glucose parameters, lipid levels, or systolic‐BP at the end of pregnancy with risk of either SGA or LGA at birth (Table 6).

**Table 6:** Multivariable‐adjusted odds ratio and 95% confidence interval, for the associations of maternal cardiometabolic markers in the third trimester, and birth outcomes.

	Birth weight				Birth HC			
	SGA		LGA		SGA		LGA	
Cardiometabolic markers	N (%)	OR (95% CI)	N (%)	OR (95% CI)	N (%)	OR (95% CI)	N (%)	OR (95% CI)
Triglycerides (mg/dL)								
Continuous (per 1‐SD increase)	19 (9.0)	0.92 (0.55, 1.52)	18 (8.5)	0.69 (0.37, 1.27)	10 (5.9)	0.77 (0.33, 1.81)	29 (17.1)	0.84 (0.52, 1.35)
Normal (< 75th Pctl)	14 (8.6)	1 Ref.	14 (8.6)	1 Ref.	8 (6.1)	1 Ref.	23 (17.4)	1 Ref.
High (≥ 75th Pctl)	5 (10.0)	1.05 (0.34, 3.24)	4 (8.0)	0.85 (0.21, 3.52)	2 (5.3)	1.09 (0.15, 8.12)	6 (15.8)	1.10 (0.33, 3.66)
Total cholesterol (mg/dL)								
Continuous (per 1‐SD increase)	19 (9.0)	1.03 (0.64, 1.67)	18 (8.5)	0.68 (0.38, 1.22)	10 (5.9)	0.72 (0.29, 1.82)	29 (17.1)	0.70 (0.44, 1.12)
Normal (< 75th Pctl)	13 (8.0)	1 Ref.	16 (9.9)	1 Ref.	10 (7.6)	1 Ref.	25 (18.9)	1 Ref.
High (≥ 75th Pctl)	6 (12.0)	1.85 (0.57, 5.75)	2 (4.0)	0.26 (0.05, 1.36)	0 (0.0)	0.00 (0.00, 0.00)	4 (10.5)	0.41 (0.11, 1.44)
LDL‐c (mg/dL)								
Continuous (per 1‐SD increase)	19 (9.0)	1.15 (0.71, 1.86)	18 (8.5)	0.70 (0.38, 1.28)	10 (5.9)	0.87 (0.33, 2.24)	29 (17.1)	0.72 (0.43, 1.20)
Normal (< 75th Pctl)	13 (8.0)	1 Ref.	15 (9.3)	1 Ref.	10 (7.6)	1 Ref.	24 (18.2)	1 Ref.
High (≥ 75th Pctl)	6 (12.0)	1.83 (0.59, 5.69)	3 (6.0)	0.62 (0.16, 2.46)	0 (0.0)	0.00 (0.00, 0.00)	5 (13.2)	0.70 (0.21, 2.33)
HDL‐c (mg/dL)								
Continuous (per 1‐SD increase)	19 (9.0)	0.97 (0.58, 1.60)	18 (8.5)	1.04 (0.60, 1.79)	10 (5.9)	1.19 (0.55, 2.60)	29 (17.1)	1.01 (0.64, 1.58)
Normal (< 75th Pctl)	14 (8.8)	1 Ref.	13 (8.1)	1 Ref.	7 (5.5)	1 Ref.	23 (18.0)	1 Ref.
High (≥ 75th Pctl)	5 (9.6)	1.36 (0.44, 4.24)	5 (9.6)	1.29 (0.38, 4.44)	3 (7.1)	2.31 (0.38, 14.06)	6 (14.3)	0.82 (0.27, 2.45)
Glucose (mg/dL)								
Continuous (per 1‐SD increase)	19 (9.0)	0.96 (0.59, 1.56)	18 (8.5)	1.17 (0.68, 2.01)	10 (5.9)	0.73 (0.33, 1.61)	29 (17.1)	0.94 (0.58, 1.51)
Normal (< 75th Pctl)	14 (9.1)	1 Ref.	10 (6.5)	1 Ref.	7 (5.8)	1 Ref.	17 (14.2)	1 Ref.
High (≥ 75th Pctl)	5 (8.6)	0.90 (0.29, 2.81)	8 (13.8)	2.49 (0.78, 7.96)	3 (6.0)	1.06 (0.17, 6.81)	12 (24.0)	1.54 (0.59, 3.99)
HOMA‐IR								
Continuous (per 1‐SD increase)	19 (9.0)	1.11 (0.65, 1.87)	18 (8.5)	0.91 (0.48, 1.70)	10 (5.9)	1.23 (0.52, 2.96)	29 (17.1)	0.89 (0.47, 1.67)
Normal (< 75th Pctl)	12 (7.5)	1 Ref.	13 (8.1)	1 Ref.	7 (5.1)	1 Ref.	25 (18.1)	1 Ref.
High (≥ 75th Pctl)	7 (13.7)	1.68 (0.58, 4.88)	5 (9.8)	1.12 (0.32, 3.89)	3 (9.4)	1.84 (0.27, 12.39)	4 (12.5)	0.57 (0.15, 2.17)
SBP (mm Hg)								
Continuous (per 1‐SD increase)	21 (11.1)	1.36 (0.79, 2.34)	19 (10.1)	0.65 (0.35, 1.19)	10 (6.3)	1.98 (0.70, 5.58)	30 (18.8)	0.76 (0.45, 1.26)
Normal (< 75th Pctl)	15 (10.6)	1 Ref.	15 (10.6)	1 Ref.	7 (5.8)	1 Ref.	23 (19.0)	1 Ref.
High (≥ 75th Pctl)	6 (12.8)	1.82 (0.57, 5.74)	4 (8.5)	0.41 (0.10, 1.69)	3 (7.7)	6.27 (0.75, 52.64)	7 (17.9)	0.58 (0.18, 1.80)
DBP (mm Hg)								
Continuous (per 1‐SD increase)	21 (11.1)	2.09 (1.22, 3.58)*	19 (10.1)	0.61 (0.34, 1.11)	10 (6.3)	2.56 (1.13, 5.81)*	30 (18.8)	0.74 (0.44, 1.25)
Normal (< 75th Pctl)	10 (7.5)	1 Ref.	14 (10.4)	1 Ref.	6 (5.4)	1 Ref.	23 (20.5)	1 Ref.
High (≥ 75th Pctl)	11 (20.0)	3.54 (1.20, 10.42)*	5 (9.1)	0.58 (0.17, 2.02)	4 (8.3)	3.34 (0.67, 16.47)	7 (14.6)	0.67 (0.22, 2.07)

*Note:* Logistic regression models were used to calculate the Odds Ratio (OR) and 95% confidence interval (95% CI). Adjusted for age categories (< 25 (ref.), 25–29, ≥ 30 years), physical activity tertile (T1: ≤ 1070 (ref.), T2:1071‐3335, T3: ≥ 3336 METs‐min/week), Mediterranean diet score tertile (T1: ≤ 8 (ref.), T2: 9‐11, T3: ≥ 12 points), GWG (insufficient (ref), adequate, excessive), BMI categories (normal weight (ref.), overweight/obesity), parity (nulliparous (ref.), multiparous), educational level (low/medium (ref.), high), smoking status (never/former smoker (ref.), current smoker), planned pregnancy (no (ref.), yes), social class (low/medium (ref.), high), sex infant. Values in bold indicate statistically significant estimates; * denotes *p* < 0.05.

Abbreviations: DBP, diastolic blood pressure; HC, head circumference; HDL‐c, high density lipoprotein cholesterol; HOMA‐IR, homeostatic model assessment for insulin resistance; LDL‐c, low density lipoprotein cholesterol; SBP, systolic blood pressure; SD, standard deviation.

## 4 Discussion

This prospective cohort study of metabolically healthy pregnant women in the Mediterranean region found that maternal lipid levels—specifically higher triglycerides and LDL‐c in early pregnancy—were positively associated with greater birthweight and an increased risk of delivering a LGA newborn. In contrast, moderately elevated diastolic‐BP throughout pregnancy was linked to a higher risk of SGA, both in terms of birthweight and HC.

Our findings align with previous research showing that maternal triglycerides, TC, and LDL‐c levels progressively increase during uncomplicated pregnancies, while HDL‐c peaks in mid‐pregnancy (Bashir et al. 2023; Nelson et al. 2010). One of the key findings in our study is that even slightly elevated maternal triglyceride levels within the physiological range during early pregnancy, but not late pregnancy, among apparently healthy pregnant women are associated with fetal overgrowth and could play a potential role in the etiology and primary prevention of macrosomia. This supports the notion that maternal triglycerides may serve as an important energy source during this sensitive and critical period of early embryonic development (Murphy et al. 2006). Our observation is reinforced by the Generation R study in Rotterdam, which found a positive association between triglyceride levels to embryonic size in early pregnancy (Gootjes et al. 2022). Elevated triglycerides in the first trimester have been consistently associated with increased birthweight and may predict LGA outcomes in normal pregnancies within Asian (Liang et al. 2018; S. M. Zhu et al. 2022), European (Adank et al. 2020; Vrijkotte et al. 2012), and North American pregnant populations (Boghossian et al. 2017; Misra et al. 2011; Omaña‐Guzmán et al. 2024). Similarly, other studies in Japan and Italy, have also confirmed that maternal hypertriglyceridemia at mid‐pregnancy, defined as the 75th cut‐off point, was associated with increased neonatal birthweight and higher risk of LGA (Di Cianni et al. 2005; Kitajima et al. 2001). Interestingly, while previous studies identified higher triglyceride cut‐offs, such as over 203 (Di Cianni et al. 2005), and 259 mg/dL (Kitajima et al. 2001), our findings show that even a lower first‐trimester threshold, over 105 mg/dL, independently predicts the likelihood of an infant being born LGA. Since elevated triglycerides in early pregnancy may predict a more severe hyperlipidemia later in pregnancy, our selected cut‐off could be helpful for screening high‐risk pregnant women early and be critical for predicting higher risks of fetal overgrowth.

Unlike earlier studies reporting an association between third‐trimester triglycerides and excessive birthweight and LGA risk (Boghossian et al. 2017; Di Cianni et al. 2005; Emet et al. 2013; Jin et al. 2016; Omaña‐Guzmán et al. 2024), we found no significant effect. Of note, with few exceptions (Di Cianni et al. 2005), these studies (Boghossian et al. 2017; Emet et al. 2013; Jin et al. 2016; Omaña‐Guzmán et al. 2024) reported mean third‐trimester triglyceride values ranging from ~245 to 555 mg/dl. In this context, Jin et al. (Jin et al. 2016) indicated that, in nondiabetic pregnant women, the optimal cut‐off points proposed by ROC curve analysis for third‐trimester triglycerides in predicting LGA infants was 3.53 mmol/L (312.7 mg/dL). Notably, the median triglyceride value during this period in our cohort was 189 mg/dL (interquartile range: 130–240 mg/dL), which likely explains the lack of observed association. Similarly, defining elevated triglycerides as the 75th cutpoint (> 240 mg/dL) for our sample did not impact the results. Based on these findings, we could postulate that only very high triglyceride values, exceeding 250 mg/dL in late pregnancy, may be linked to newborn size. This argument aligns with the USA's National Lipid Association recommendation that triglyceride levels should not exceed 250 mg/dl at any time during pregnancy to avoid obstetrical complications (Jacobson et al. 2015).

Fetal growth is influenced by maternal lipid metabolism (Mulder et al. 2024), yet the mechanisms linking early pregnancy triglycerides to fetal overgrowth remain largely unknown, as maternal triglycerides do not directly cross the placenta. It is hypothesized that placental lipoprotein lipase and other lipases break down triglycerides early in pregnancy, releasing free fatty acids for placental uptake and delivery to the fetus. These fatty acids are then used by trophoblast cells to meet metabolic demands, produce essential hormones, and support fetal development. Thus, excessive mother‐to‐fetus fatty acid transfer could contribute to abnormal growth patterns, including overgrowth and lipid accumulation in fetal tissues (Duttaroy and Basak 2022; Mulder et al. 2024).

In pregnancy, as in the nonpregnant state, circulating triglyceride levels enrich VLDL at low concentrations and LDL‐c at all concentrations (Mulder et al. 2024). This may explain our observed positive association between first‐trimester LDL‐c levels and larger birthweight, which is consistent with the role of triglyceride‐rich lipoproteins in transporting fatty acids for placental uptake and fetal development. Similar associations have been reported in specific populations, such as women with pre‐eclampsia (Boghossian et al. 2017) or underweight status (S. M. Zhu et al. 2022), and in late pregnancy among women in rural Gambia, a low‐income setting (Okala et al. 2020). However, these findings contrast with larger‐scale evidence. A 2018 meta‐analysis encompassing 46 studies, including trimester‐specific analyses, concluded that maternal LDL‐c levels (*n* = 18 studies) across pregnancy were not significant causative factors of large birthweight outcomes, despite some heterogeneity in the findings (J. Wang et al. 2018). Similarly, a recent meta‐analysis by Mahindra et al. (Mahindra et al. 2021), which included 12 studies focusing exclusively on metabolically healthy women without confounding conditions like obesity, hypertension, or gestational diabetes—factors known to influence lipid levels—also found no significant effect of LDL‐c or VLDL‐c on LGA from early to late pregnancy. The authors concluded that these lipids are not predictive markers of fetal overgrowth in normal pregnancies. In accord with previous research, we did not detect any association for LDL‐c in the third trimester. However, it is worth noting that standard LDL‐c measurements may not fully capture the complexity of lipid‐related metabolic risk. A more specific study focusing on changes in LDL‐c subfractions (total particle concentration and size) throughout gestation in normal pregnancy could clarify whether more subtle or functionally distinct lipid alterations are involved in fetal growth (Rideout et al. 2022). This is especially relevant because individuals, even with equivalent LDL‐c levels, can differ significantly in their LDL particle profiles, which may have distinct biological implications (Mora et al. 2009). Thus, the lack of association in our study might partly reflect this limitation, and future research employing advanced lipoprotein profiling is needed to further explore this relationship.

Aligned with some previous research (Adank et al. 2020; Emet et al. 2013; Gootjes et al. 2022; Jin et al. 2016; Omaña‐Guzmán et al. 2024; S. M. Zhu et al. 2022), we did not find any relationship between maternal HDL‐c levels during pregnancy and birthweight‐related outcomes. However, the literature on this topic remains conflicting. It has been demonstrated in previous research that elevated HDL‐c levels in early to mid‐pregnancy are associated with a reduced risk of low birthweight (LBW, < 2500 g) (Okala et al. 2020) and SGA among overweight/obese women (Bever et al. 2020). Also, some researchers have revealed that higher HDL‐c concentrations, especially in the second and third trimesters, are associated with a lower risk of LGA (Bever et al. 2020)/macrosomia (Clausen et al. 2005; Jin et al. 2016). This finding aligns with the notion that HDL‐c protects against cardiovascular risk via its inherent antioxidative, anti‐inflammatory, and immunomodulatory properties (Woollett et al. 2022). By contrast, other authors have pointed that HDL‐c levels throughout pregnancy, particularly in the third trimester, are inversely correlated with newborns' birth weight (Boghossian et al. 2017; Misra et al. 2011; H. Wang et al. 2020; J. Wang et al. 2018) and positively rather than negatively associated with the risk of SGA (H. Wang et al. 2020; J. Wang et al. 2018); the stronger associations appear to occur among pregnant women with pre‐pregnancy overweight/obesity or gestational diabetes (J. Wang et al. 2018). One proposed explanation involves placental dysfunction, which may impair HDL‐c transport to the fetus, raising maternal HDL‐c levels and ultimately leading to SGA; argument supported by findings of reduced cord blood HDL levels in fetal growth restriction cases (Kwiterovich et al. 2005). All these divergences make it difficult to draw any firm conclusions about such a relationship. Meanwhile, our results highlight the importance of lipid screening and prevention during early pregnancy, as prenatal monitoring, to potentially improve birthweight outcomes and mitigate risks.

One striking and unexpected finding from our study was the lack of association between fasting glucose levels in either the first or third trimester and neonatal size at birth. This contrasts with prior research showing positive associations between glucose levels—fasting or postprandial—during pregnancy and birthweight or LGA risk, even without impaired glucose tolerance or overt diabetes, with mean glucose levels ranging from 75 to 88 mg/dL (Geurtsen et al. 2019; Guo et al. 2021; Voldner et al. 2010; Yang et al. 2023; Zhao et al. 2023; Zou et al. 2022). In our relatively healthy population, fasting mean glucose levels of 70 mg/dL in early pregnancy and 68 mg/dL in late pregnancy fall within the low‐normal range, which might have reduced associations toward the null. Consequently, our data did not allow us to research the impact of elevated fasting glycemia levels or extreme hyperglycemia. Similarly, we could not find an association between IR during pregnancy and fetal growth outcomes, as also reported by others (Akinola et al. 2024; Bomba‐Opon et al. 2009; Voldner et al. 2010). This finding might be partially explained by the indirect HOMA‐IR method used. A more specific study using the euglycemic hyperinsulinemic clamp—the gold standard for directly measuring IR (DeFronzo et al. 1979)—could shed light on this poorly studied and inconclusive relationship, particularly in populations with low‐normal glucose level (Akinola et al. 2024; Bomba‐Opon et al. 2009; Tanaka et al. 2018; Voldner et al. 2010; Yamashita et al. 2014).

Our study found that elevated diastolic‐BP in late pregnancy was linked to a higher risk of SGA infants in terms of birthweight. Importantly, this association was independent of important confounders such as GWG, diet, PA, as these are determinants capable of modifying both maternal cardiometabolic health and fetal growth, as it has been reported (Badon et al. 2018; Flor‐Alemany et al. 2021). This finding not only confirms previous reports on severe essential hypertension and pre‐eclampsia (Bakker et al. 2011; Xiong and Fraser 2004; B. Zhu et al. 2019), but also expands on them by focusing on a cohort of healthy Mediterranean women with uncomplicated pregnancies and BP well below the threshold for hypertensive disorders of pregnancy (BP < 140/90 mmHg). Regarding this, a large UK study (*n* = 9697) involving normotensive pregnant women with repeat antenatal blood pressure measurements, showed that higher systolic‐BP in the first trimester and greater increases in both systolic and diastolic‐BP from mid to late pregnancy were associated with lower birthweight and SGA (Macdonald‐Wallis et al. 2014). Similarly, a Swedish study in non‐hypertensive women also found that prehypertension (diastolic BP 80–89 mmHg) at 36 gestational weeks predicted an increased risk of SGA (Wikström et al. 2016). Furthermore, it was found that fetuses of Mexican mothers with high systolic‐BP and diastolic‐BP had lower weight in the 6th month, and a higher diastolic‐BP trajectory within the normal range throughout pregnancy was associated with lower birthweight (Omaña‐Guzmán et al. 2024). Consistent with some studies (Bakker et al. 2011; Omaña‐Guzmán et al. 2024; Wikström et al. 2016), we identified diastolic‐BP as a stronger predictor of lower birthweight than systolic‐BP. Reinforcing this, a small study also showed that among healthy normotensive pregnant women, elevated diastolic‐BP during mid‐to‐late pregnancy, as measured by 24‐h ambulatory BP monitoring—often considered the gold standard—was the predominant risk factor for low birthweight, whereas systolic‐BP levels were not (Churchill et al. 1997). In this regard, evidence specifically aimed at comparing systolic and diastolic‐BP measurements during pregnancy, and their association with infant birthweight, suggests that diastolic‐BP may better reflect increases in maternal peripheral vascular resistance, as it is less influenced by moment‐to‐moment stimuli than systolic‐BP (Churchill et al. 1997; Iwama et al. 2016). This could partly explain these discrepant results, but the real causes remain unknown. Additionally, caution is needed when comparing BP‐related outcomes across cohorts, as differences in measurement methods (e.g., electronic vs. auscultatory), settings, and timing can introduce variability in BP values and their associations with neonatal outcomes.

Importantly, our study also identified a robust relationship between higher maternal diastolic‐BP and the risk of SGA based on HC, both when measured in early and late pregnancy. This finding is significant as smaller HC has been linked to future neurological and cognitive challenges, as previously reported (Abdelmageed et al. 2024; Noda et al. 2021; Gampel and Nomura 2014). One proposed mechanism underlying these associations is that even mildly elevated BP may disrupt the development of the placental villous tree, reducing its functional capacity to transfer oxygen and nutrients to the fetus, thereby restricting growth (Kingdom et al. 2000). Taken together, our study highlight the critical importance of careful BP monitoring throughout pregnancy, even in normotensive women, as essential part of perinatal care.

This longitudinal study, which spans from early pregnancy to delivery, presents several strengths. Utilizing a large data set from a well‐characterized cohort, it benefited from rigorous prospective data collection and critical maternal information, which, by adjusting for multiple covariates, minimized potential confounding effects and allowed for more robust conclusions. It is worth noting that, despite encouraging findings from prior studies exploring similar associations, a major concern remains: with few exceptions (Bakker et al. 2011; Clausen et al. 2005; Liang et al. 2018; Voldner et al. 2010; Vrijkotte et al. 2012; B. Zhu et al. 2019), most research did not adjust for key confounders like diet quality, physical activity, and/or weight gain, potentially compromising the validity of their estimates; adopting and maintaining healthy lifestyle behaviors would likely reduce cardiometabolic risk during pregnancy (Flor‐Alemany et al. 2021) and promote normal fetal growth (Badon et al. 2018). However, this is only conjecture, and further research is needed that controls for such important factors.

Notably, it is among the first studies to analyze the effects of a wide range of maternal cardiometabolic markers on neonatal growth across two critical time points during pregnancy. Our study expands prior evidence by focusing on a Mediterranean cohort of metabolically healthy women with uncomplicated pregnancies. Additionally, we also evaluated HC at birth, not just birthweight, which is typically the only parameter analyzed in most existing literature. Our study also has limitations. First, the cardiometabolic markers were assessed only once per trimester, potentially affecting their reliability due to fluctuations. Second, it's important to note that BP measurements were not acquired through 24‐h ambulatory monitoring. Instead, they were taken during routine antenatal visits and therefore have inherent interobserver variability and measurement timing‐related variability, which could influence our findings. Third, as this is an observational study, causality cannot be claimed. And lastly, the possibility of residual confounding cannot be ruled out.

## 5 Conclusions

In summary, this prospective cohort study found significant associations between maternal metabolic biomarkers in the first and third trimesters of pregnancy and newborn size. Elevated triglyceride and LDL‐c levels at the beginning of pregnancy were positively associated with higher birthweight and an increased risk of LGA for weight. Additionally, BP during pregnancy—particularly diastolic‐BP—was independently associated with an increased risk of SGA based on both weight and HC at birth. Based on our findings, it would be beneficial to provide health education before pregnancy and to screen women early for lipid and blood pressure disorders as well as actively monitor these maternal cardiometabolic risk factors throughout pregnancy to ensure normal fetal development and the future health of the child.

## Author Contributions

Conceptualization: Ehsan Motevalizadeh, Andrés Díaz‐López, and Victoria Arija. Data curation: Victoria Arija, Cristina Jardí. Methodology: Victoria Arija. Resources: Victoria Arija. Formal analysis: Ehsan Motevalizadeh, Andrés Díaz‐López Investigation: Ehsan Motevalizadeh, Andrés Díaz‐López, Cristina Jardí, Cristina Rey‐Reñones, F.M. Validation: Victoria Arija. Writing – original draft preparation: Ehsan Motevalizadeh, and Andrés Díaz‐López Writing – review and editing: Ehsan Motevalizadeh, Andrés Díaz‐López, and Victoria Arija. Supervision: Victoria Arija. Project administration: Victoria Arija. Funding acquisition: Victoria Arija. All authors have read and agreed to the published version of the manuscript.

## Consent

Informed consent was obtained from all participants included in the study.

## Conflicts of Interest

The authors declare no conflicts of interest. The funders had no role in the design of the study; in the collection, analyses, or interpretation of data; in the writing of the manuscript; or in the decision to publish the results.

## Data Availability

The datasets generated and/or analyzed during the current study are not publicly available due to subject confidentiality but are available from the corresponding author on reasonable request.
